# Open Gastrostomy by Mini-Laparotomy: Our Method

**DOI:** 10.7759/cureus.45506

**Published:** 2023-09-18

**Authors:** Shuichi Ishibashi, Koji Kumori, Junko Manako, Narimasa Funabashi, Yoko Senaha, Masaaki Hidaka

**Affiliations:** 1 Digestive and General Surgery, Shimane University Faculty of Medicine, Izumo, JPN

**Keywords:** peg, lag, pimd, mini-laparotomy, children, gastrostomy

## Abstract

When percutaneous endoscopic gastrostomy (PEG) is not feasible owing to anatomical obstacles, laparotomic or laparoscopic gastrostomy (LAG) is an alternative. At our institution, LAG has been the first choice for patients who are unable to undergo PEG; however, we have introduced a small open gastrostomy through a 2-cm-long transverse incision since 2020. By December 2022, 12 patients had undergone this procedure without complications. In one case where the stomach was located cephalad to the rib arch and the patient had a round dorsum, the incision wound was extended, and a lengthy operation was required. We believe that our small-incision gastrostomy is a useful option in addition to LAG for cases in which PEG is difficult to perform. Further studies are required to determine the indications for this procedure.

## Introduction

Gastrostomy tube feeding provides a way for patients who do not receive sufficient energy and nutrients through oral feeding to receive long-term enteral nutrition. It is important to consider a patient's physical characteristics, such as scoliosis and a stomach located above the costal arch, when selecting a surgical method, especially for individuals with profound intellectual and multiple disabilities (PIMD) [1]. In our department, laparoscopic gastrostomy (LAG) has typically been the go-to procedure when we encounter cases in which percutaneous endoscopic gastrostomy (PEG) may not be safe. This is especially true when the stomach is positioned cranial to the costal arch or when the risk of vascular puncture is high due to axial torsion. However, since 2020, open gastrostomy by mini-laparotomy (M-OG) has been adopted as an alternative to LAG for both adults and children. This procedure involves a transverse incision of 2 cm in length and is selected over LAG in certain cases. We herein discuss the performance of M-OG in our department and address the concerns identified during the operation.

## Materials and methods

Patients who underwent M-OG in our department between January 2020 and December 2022 were enrolled in this study. Patients who underwent other operations were excluded. Patients who underwent LAG between January 2011 and December 2019 were included for comparison. Student's t-test was used for statistical analysis, with a significance level of ≦5%. Institutional review board (IRB) (Medical Research Ethics Committee of Shimane University Faculty of Medicine) approval was granted on March 29, 2023 (approval number: 6820). We provided the patients in this research with the opportunity to opt out.

**Figure 1:**
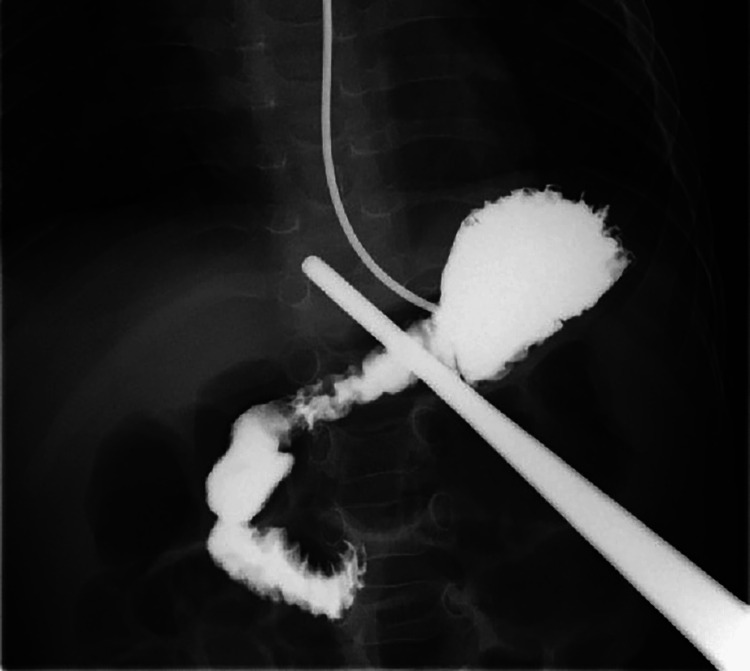
Upper gastrointestinal imaging The forceps is located at the left costal arch.

**Figure 2:**
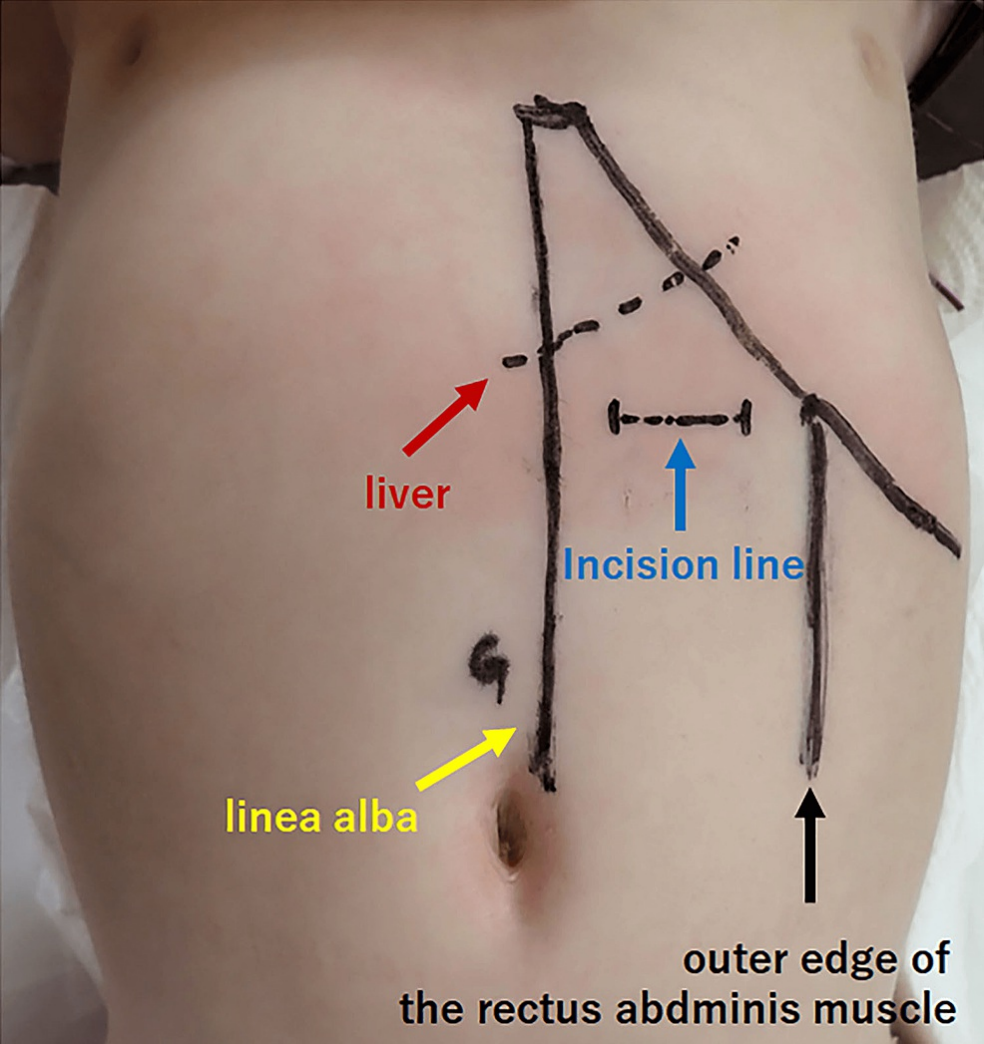
Marking before the operation

**Figure 3:**
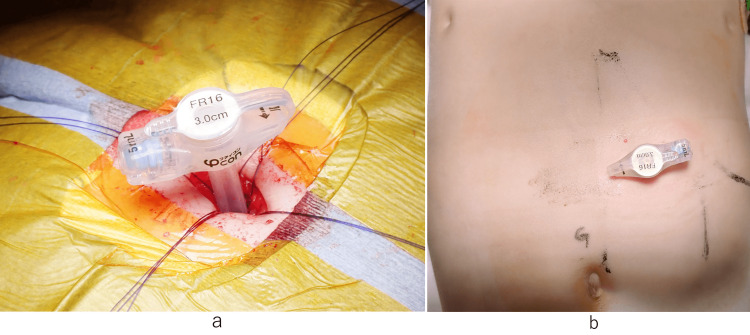
Open gastrostomy by mini-laparotomy a: After the gastrostomy tube is inserted, it is important to secure the abdominal wall and stomach at four points. b: Postoperative image.

We introduce a specific technique for M-OG. Before surgery, upper gastrointestinal imaging is performed to determine the position of the stomach and check for torsion. At the same time, a 24-hour pH monitor test is conducted to confirm the presence or absence of gastroesophageal reflux (Figure 1). After inducing general anesthesia, ultrasound (US) is used to locate and mark the position of the stomach and its relationship with the liver, linea alba, and outer edge of the rectus abdominis muscle. The location below the left costal arch is also determined (Figure 2). To perform gastrostomy, we recommend using a button-type tube with an outer diameter of 16 Fr. The shaft length should be selected based on the distance from the body surface to the peritoneum, plus 1.5 cm. A 2-cm horizontal incision is made at the designated location during surgery. The abdominal cavity is observed, and the stomach is pulled toward the incision. Next, a purse-string suture is placed on the retracted stomach, and a button-type gastrostomy tube is inserted into the center. The purse-string suture is closed once the tube is in place. To secure the abdominal wall, the gastric wall, peritoneum, and posterior sheath of the rectus abdominis muscle are fixed at four points. Finally, the wound is closed (Figure 3).

## Results

**Figure 4:**
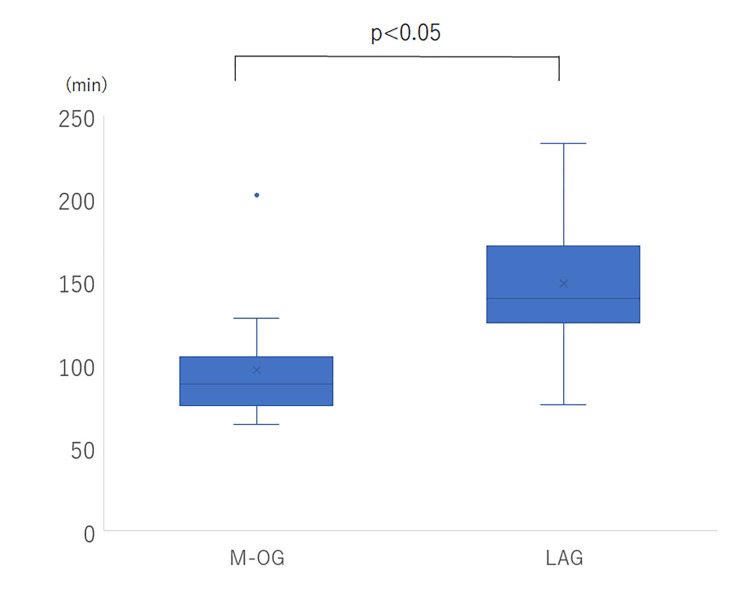
Operative time (M-OG versus LAG) M-OG: open gastrostomy by mini-laparotomy, LAG: laparoscopic gastrostomy

The present study included 12 patients who underwent M-OG and 32 who underwent LAG. The mean patient age was 25.5 years in the M-OG cohort (range: 0-76 years) and 33.4 years in the LAG cohort (range: 5-72 years) (p=0.28). Of those undergoing M-OG, 58.3% were female, while 50% were female in the LAG cohort (p=0.74). There was also no difference in the mean patient weight between the two groups (34.8 kg (M-OG) versus 33.4 kg (LAG), p=0.11). Table 1 and Table 2 show the age at the time of surgery and background diseases for each surgical procedure. The primary reason for gastrostomy was swallowing in coordination/motility disorder. The median operative time for M-OG was 99.1 minutes (range: 64-202 minutes), which was significantly shorter than that for LAG (148.6 minutes) (p<0.0001), as depicted in Figure 4. There were no complications, and all patients received nutrition infusion through the gastrostomy on the first day after the operation, with an uncomplicated course.

**Table 1:** Demographic characteristics of the patients according to surgical techniques M-OG: open gastrostomy by mini-laparotomy, LAG: laparoscopic gastrostomy

	M-OG (n=12)	LAG (n=32)	p-value
Mean age (years)	25.5 (range: 0-76)	33.4 (range: 5-72)	0.28
Female (%)	58.3	50	0.74
Mean weight (kg)	34.8 (range: 4.6-47.8)	33.4 (range: 9-58.9)	0.11

**Table 2:** Summary of the diagnoses M-OG: open gastrostomy by mini-laparotomy, LAG: laparoscopic gastrostomy

M-OG (n=12)		LAG (n=32)	
Cerebral palsy	5 (42%)	Cerebral palsy	17 (52%)
Duchenne’s muscular dystrophy	2 (16%)	Duchenne’s muscular dystrophy	8 (25%)
Dravet syndrome	1 (8%)	Facioscapulohumeralatrophy	1 (3%)
Hypochondroplasia	1 (8%)	Subarachnoid hemorrhage	1 (3%)
Intracerebral hemorrhage	1 (8%)	Cornelia de Lange syndrome	1 (3%)
Lennox-Gastaut syndrome	1 (8%)	Huntington's disease	1 (3%)
Mitochondrial disease	1 (8%)	Others	3 (9%)

**Figure 5:**
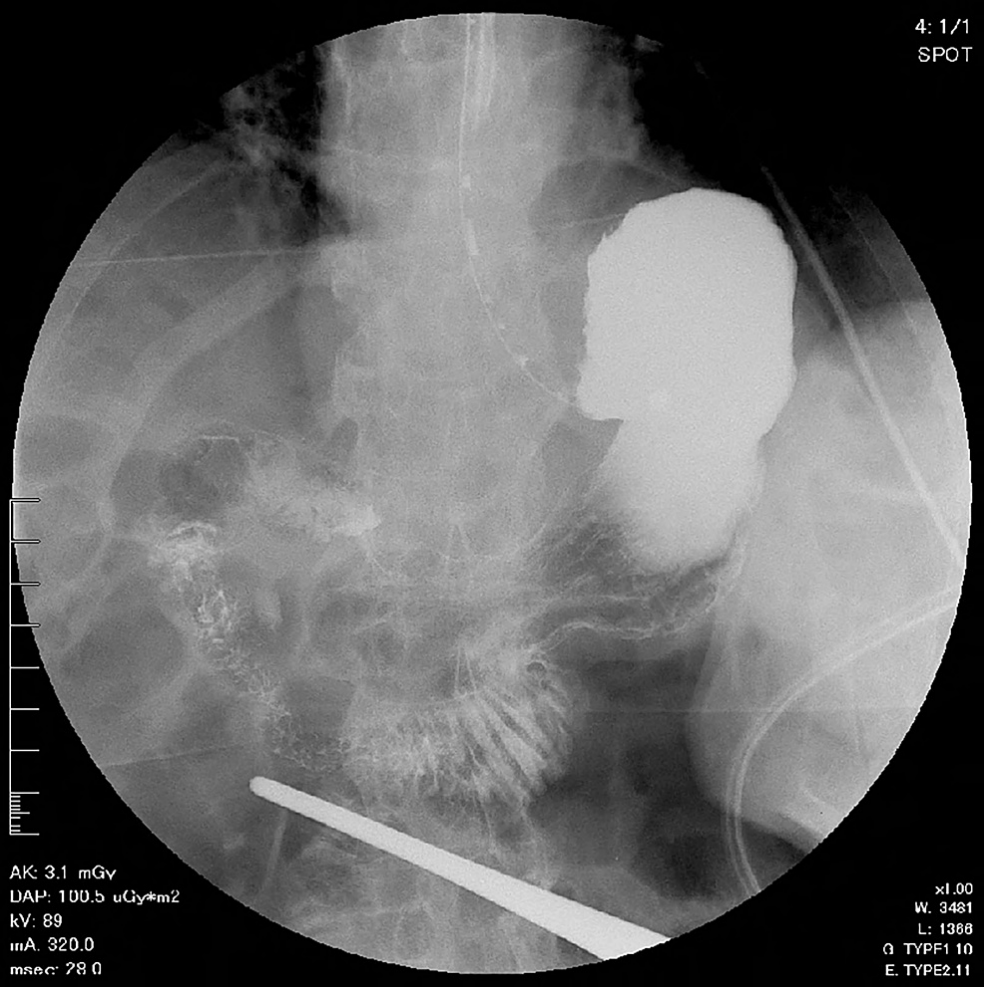
Upper gastrointestinal imaging of a patient in whom the operative time was 202 minutes The stomach showed signs of long-axis torsion and was positioned along the duodenum, above the costal arch.

In one case, the procedure took 202 minutes to complete. The patient was a 76-year-old male with a hunched back and long-axis gastric volvulus. The entire stomach and duodenum were positioned above the costal arch (Figure 5). Owing to the patient's thick subcutaneous fat and limited gastric mobility, it took some time to identify the surgical site, which resulted in an extended operation time.

## Discussion

A gastrostomy is typically performed to administer nutritional supplements when oral intake is hindered by issues such as central nervous system disease or decreased swallowing function. It can also be used when patients have difficulty in nasogastric tube feeding or self-extraction. In situations where oral intake is possible, but the necessary amount of food and water cannot be consumed, or when the nasogastric tube hinders swallowing, removal of the nasogastric tube can improve swallowing [2]. Enteral nutrition through gastrostomy is a safer option than parenteral nutrition, which involves intravenous delivery. This is because there is a lower risk of complications (e.g., hyperglycemia and sepsis) [3]. In our department, many patients require gastrostomy because of difficulties with oral intake. Additionally, changing the injection route is sometimes necessary to avoid the long-term placement of a nasogastric tube. This is because nasogastric tube syndrome can occur, leading to vocal cord paralysis during insertion. Direct placement of the nasogastric tube can also cause infection and necrosis, as well as bilateral vocal cord paralysis due to pressure on the posterior cricoarytenoid muscle [4]. Placement of a nasogastric tube for extended periods is generally not recommended. Instead, gastrostomy is typically performed to administer injectable nutrition. If a patient experiences abdominal distension due to compression, gastrostomy may be recommended for decompression. This is because swallowing excessive amounts of air can lead to ileus, which can cause intestinal dilation and ultimately require surgical intervention [5].

Surgical gastrostomy was first successfully performed in the 1870s. Two gastrostomy methods, the Witzel method and the Stamm method, were reported in the 1890s and are still used as gastrostomy procedures [6,7]. Furthermore, the PEG and LAG procedures are gaining popularity because of their less invasive nature [8-10]. However, PEG has limitations in terms of puncture and construction sites due to its inability to guide the endoscope during surgery, especially in patients with trunk deformation. Additionally, a small stomach capacity coupled with poor positioning of the gastrostomy is associated with a significant risk of passage obstruction. In contrast, LAG is associated with benefits such as lower risks and better patient outcomes [9-11]. However, a study conducted by a single institution revealed that patients who underwent LAG for gastrostomy experienced a higher incidence of bleeding, erythema, swelling, and granulation proliferation than those who underwent open surgery [12]. Our department has successfully performed M-OG procedures with a 2-cm horizontal incision in numerous cases. We prioritized the identification of the ideal position for placement before the operation, considering factors such as the position and shape of the stomach and its relationship with other organs after the induction of general anesthesia. This approach ensured optimal results for our patients.

The operation that took 202 minutes was challenging because the stomach and duodenum were positioned above the costal arch and had limited mobility. To prevent similar difficulties in the future, we devised a method that involves using a camera and forceps to observe the entire stomach through a small incision on the skin. This method, which we have successfully applied in our department, involves single-incision laparoscopic-assisted appendectomy using a Wrap Protector^TM^ and E･Z Access® with two ports [13]. We aimed to reduce the operation time by checking the stomach's mobility and determining the best position for the operation.

There are two main types of gastrostomy tubes: balloon and bumper types (classified based on the shape of the internal stopper in the stomach) and button and tube types (classified based on the shape outside the abdominal wall). These types can be combined to create four different classifications: balloon buttons, balloon tubes, bumper buttons, and bumper tubes. In our department, balloon-button-type tubes are used in nearly all situations. This is because the "balloon type" causes less discomfort for patients during insertion and removal for replacement, while the "button type" has fewer exposed parts, leading to fewer accidental removals by the patient or caregiver. In addition, because of its concealed placement under clothing, the button-type tube has a more aesthetically pleasing appearance [14]. Using a balloon-button-type tube during the initial insertion simplifies the injection process for families. However, determining the appropriate shaft length can be challenging, and it must be replaced more frequently in comparison to the bumper-type option.

The limitation of the study is its retrospective design. The smaller sample size characteristic of a single-center study may miss small differences. Further studies on this topic should be conducted to ensure more consistent results.

## Conclusions

Performing M-OG in our department has proven to be a valuable alternative to LAG in gastrostomy cases where PEG is not feasible. However, in complex scenarios, such as those mentioned above, it might be more beneficial to use a camera and forceps through the skin incision to observe the entire abdominal cavity and locate the gastrostomy site. Our goal was to gather more cases and establish clear guidelines for the selection of an appropriate surgical approach.
